# Diagnostic Efficacy of Cervical Elastography in Predicting Spontaneous Preterm Birth in Pregnancies with Threatened Preterm Labor †

**DOI:** 10.3390/diagnostics15151934

**Published:** 2025-07-31

**Authors:** Hayan Kwon, Ji-Hee Sung, Hyun Soo Park, Ja-Young Kwon, Yun Ji Jung, Hyun-Joo Seol, Hyun Mi Kim, Won Joon Seong, Han Sung Hwang, Soo-Young Oh, on behalf of The Korean Consortium for the Study of Cervical Elastography in Prediction of Preterm Delivery

**Affiliations:** 1 Department of Obstetrics and Gynecology, Yonsei University Health System, Institute of Women’s Life Medical Science, Yonsei University College of Medicine, Seoul 03722, Republic of Korea; whitekwon@yuhs.ac (H.K.);; 2 Department of Obstetrics and Gynecology, Samsung Medical Center, Sungkyunkwan University School of Medicine, Seoul 06351, Republic of Korea; jihee.sung@samsung.com; 3 Family Medicine Residency, Providence St. Joseph Eureka Hospital, Eureka, CA 95501, USA; 4 Department of Obstetrics and Gynecology, Korea University Guro Hospital, Korea University College of Medicine, Seoul 08308, Republic of Korea; 5 Department of Obstetrics and Gynecology, School of Medicine, Kyungpook National University Hospital, Kyungpook National University, Daegu 41404, Republic of Korea; 6 Department of Obstetrics and Gynecology, Konkuk University Medical Center, Konkuk University School of Medical, Seoul 05030, Republic of Korea

**Keywords:** threatened preterm labor, elastography, preterm birth, transvaginal ultrasound, cervical length

## Abstract

**Background/Objective**: Accurately identifying women at high risk for preterm birth among those with threatened preterm labor (PTL) is crucial for effective interventions or tocolytic management to reduce preterm birth and its complications. This study aimed to determine the predictive value of cervical elastography for preterm delivery before 37 weeks of gestation in patients with threatened PTL and a cervical length greater than 15 mm. **Methods**: This prospective cohort study included pregnant women presenting with threatened PTL at between 24 and 34 weeks gestation. All participants underwent cervical elastography at diagnosis. We compared cervical elastography parameters between women who delivered spontaneously preterm (<37 weeks) and those who delivered at full term and assessed the ability of these parameters to predict spontaneous preterm delivery. **Results**: Among the 107 enrolled individuals with threatened PTL and a cervical length of ≥15 mm, 55 (42%) experienced preterm birth (<37 weeks). Internal os stiffness (IOS), internal-to-external os stiffness ratio (IOS/EOS ratio), and elasticity contrast index (ECI) were significantly associated with a risk of preterm birth compared to full-term birth. The IOS/EOS ratio was associated with 10-fold higher odds of preterm birth at <37 weeks (95% confidence interval [CI], 1.82–59.98), and ECI was associated with 1.5-fold higher odds (95% CI, 1.01–2.37). The IOS/EOS ratio demonstrated good predictive value (area under the curve (AUC) = 0.678) and the combination of CL ≤ 25 mm and the IOS/EOS ratio had good diagnostic performance for predicting preterm birth (AUC = 0.708). **Conclusions**: Cervical elastography using the E-Cervix™ system appears to improve the ability to predict preterm birth in pregnant women with threatened PTL and a cervical length greater than 15 mm.

## 1. Introduction

Preterm birth, defined as delivery before 37 weeks of gestation, remains a critical concern in obstetrics. Despite decades of research and clinical advancements, an estimated 13.4 million babies are born preterm annually, accounting for approximately 1 in 10 births worldwide [1,2,3]. This rate has been rising, partly due to increasing maternal age and the widespread use of advanced assisted reproductive technologies [4,5]. Preterm birth is associated not only with immediate neonatal morbidity and mortality but also leads to lasting long-term complications, including neurological developmental issues and cardiovascular diseases, resulting in a substantial social and economic burden. Preterm labor (PTL) is considered the most significant cause of preterm birth, contributing to an estimated 40–50% of cases [5,6].

Threatened PTL, characterized by uterine contractions occurring between 20 and 37 weeks of gestation with minimal or no cervical changes, is a leading cause of hospital admissions during pregnancy [7,8]. While threatened PTL does not always progress to preterm birth, approximately 25–30% of cases do advance to preterm delivery [9]. Beyond the direct impact of preterm births, threatened PTL itself presents a significant challenge as it is the most common cause of hospitalization during the second trimester of pregnancy. This condition often necessitates prolonged hospital stays, tocolytic treatment with potential side effects, causes significant distress for the pregnant woman and her family, and incurs high economic costs. Therefore, it is crucial to accurately distinguish between threatened PTL that can be managed conservatively and discharged, and actual PTL at higher risk of imminent delivery. Such differentiation ensures appropriate care and management, as high-risk of PTL requires timely interventions, including antenatal corticosteroids for fetal lung maturation and magnesium sulfate for neuroprotection.

Various methods have traditionally been employed to predict imminent delivery in women with threatened PTL. These include evaluating cervical status through cervical length (CL) measurement or the Bishop score, assessing biochemical markers such as fetal fibronectin or phosphorylated insulin-like growth factor binding protein 1 (phIGFBP-1), and serially assessing uterine dynamics via tocodynamometry [10,11,12,13,14]. Other trials explored uterine electromyography and uterine artery Doppler velocity as a novel approach to predict preterm birth [15,16,17]. However, none of these examinations have consistently and precisely distinguished actual PTL leading to preterm birth, primarily due to their low positive predictive values.

Cervical elastography is an emerging ultrasound technique that quantitatively assesses cervical tissue stiffness, potentially offering valuable information for predicting the risk of preterm birth. Unlike traditional CL measurement, elastography can detect subtle changes in cervical tissue stiffness or elasticity with greater sensitivity, enabling earlier prediction of preterm birth risk. As an ultrasound-based method, it is non-invasive and can be performed repeatedly at multiple time points during pregnancy, allowing for continuous monitoring of cervical status. Although standardized measurement protocols and normal reference ranges have yet to be fully established, recent studies suggest that cervical elastography may serve as a useful adjunctive tool for predicting preterm birth [18,19,20,21,22,23].

This study aims to evaluate the diagnostic efficacy of cervical elastography in predicting imminent preterm delivery by assessing cervical stiffness in women with threatened PTL. The ultimate goal is to identify high-risk pregnant women early, enabling appropriate management with preventive treatments (e.g., progesterone administration, cervical cerclage) or necessitating hospitalization for close observation.

## 2. Materials and Methods

### 2.1. Study Population

This was a prospective cohort study of women with threatened PTL who underwent transvaginal ultrasonography assessment using E-Cervix™ application between 24 weeks 0 days and 34 weeks 6 days of gestation. Threatened PTL was defined as those showing 6 or more contractions in 1 h on cardiotocography with intact membranes. Enrollment occurred at maternity center in seven referral university hospitals from June 2018 to December 2020. According to the study protocol, cervical elastography was performed simultaneously with CL measurement at the diagnosis of threatened PTL. Women without an in situ cervical cerclage at the time of enrollment or uterine anomaly were eligible and women were included regardless of progesterone use. Women with abnormal cervix shape, triplet pregnancy, placenta previa, abruptio placentae, iatrogenic preterm birth due to maternal and fetal conditions, preterm delivery within 24 h of enrollment, and pregnancies using tocolytics before CL measurements were excluded. Pregnancy outcomes as well as demographic and obstetric parameters were collected. This study was approved by the institutional review boards (IRB) of all participating institutions (IRB No. Yonsei University Severance Hospital, 1-2018-0022; Dongguk University Ilsan Hospital, 2017-12-019-007; Kangbuk Samsung Hospital, 2018-06-006; Samsung Medical Center, 2018-03-073-015; Kyung Hee University Hospital at Gangdong, 2018-03-002; Kyungpook National University Chilgok Hospital, 2018-08-005-007H and Konkuk University Medical Center, 1040070), and written informed consent was obtained from all participants prior to enrollment. We followed the ethical standards for human experimentation established in the Declaration of Helsinki. The patient agreed to publication of the elastography image of the uterine cervix.

### 2.2. Maternal Clinical Variables

After enrollment, maternal demographic and clinical data were manually abstracted from the medical record following enrollment. These data included maternal age, body mass index, prior uterine or cervical procedures, and pregnancy history. Information on management of the current pregnancy, including progesterone use, cerclage placement, and use of tocolytic agents, and delivery outcomes were obtained following enrollment. Delivery information included gestational age at time of delivery, indication for preterm deliveries, and mode of delivery. Among women with preterm delivery in this cohort, only those who had experienced spontaneous preterm delivery were considered eligible. Spontaneous preterm delivery was defined as a birth before 37 completed weeks of gestation, due either to PTL or to preterm premature rupture of membranes.

### 2.3. Cervical Length and Elastographic Measurements

After measuring the CL, cervical elastography was performed using a 5–9 MHz transvaginal transducer via ultrasound system (WS80A Ultrasound System, Samsung Medison, Seoul, Korea) with accessible E-Cervix™ software, Version 1.0 beta. According to a previously published standardized E-Cervix protocol, cervical elastography assessment was performed at least three times in the mid-sagittal plane of the cervix with automatically when the software detects lack of motion artifact [24]. Five E-Cervix parameters were obtained, including (1) internal os stiffness (IOS), defined as the strain within 1 cm of the internal os; (2) external os stiffness (EOS), defined as the strain within 1 cm of the external os; (3) internal-to-external os stiffness ratio (IOS/EOS ratio), defined as IOS divided by the EOS; (4) elasticity contrast index (ECI), defined as a measure of strain heterogeneity within the entire cervix; and hardness ratio (HR), which is the percentage of the upper 30% of the hard area within the total cervix, as well as cervical length. IOS, EOS, and ECI defined as a range between 0 (hard) and 1 (soft). ECI represented heterogeneity of tissue with higher values indicating more heterogeneous and softer tissue, and the higher HR represented the more proportion of hard tissue in cervical tissue.

### 2.4. Statistical Analysis

Maternal baseline characteristics and sonographic findings including E-Cervix parameters were compared between patients who delivered at <37 weeks of gestation (preterm delivery) and those who delivered at ≥37 weeks of gestation (term delivery). Descriptive statistics as median (min–max) or mean ± standard deviation for continuous variables and frequency (percentages) for categorical variables were calculated separately by groups. For continuous variables, Shapiro–Wilk and Kolmogorov–Smirnov tests were used to test for normal distribution of the data. Comparisons between groups were made using Student’s *t*-test for normally distributed data or the Mann–Whitney U test for non-normally distributed data. Categorical variables were analyzed using the chi-square test or Fisher’s exact test, depending on expected frequencies. To identify significant diagnostic parameters, we performed multivariable logistic regression adjusting confounders for the prediction of preterm birth. To evaluate the ability of each E-Cervix parameters and CL to predict preterm birth, receiver operating characteristic curve (ROC) curves was generated and the area under the curve (AUC) were calculated. To further assess the clinical utility of the sonographic parameters or combination of parameters for predicting preterm delivery, we identified the optimal cutoff reference point on the ROC for each parameter by calculating the Youden index, which maximizes the sensitivity and specificity of a measure. The statistical analysis was performed using SPSS version 29.0 software (SPSS Inc., Chicago, IL, USA). *p <* 0.05 was considered statistically significant.

## 3. Result

**Figure 1:**
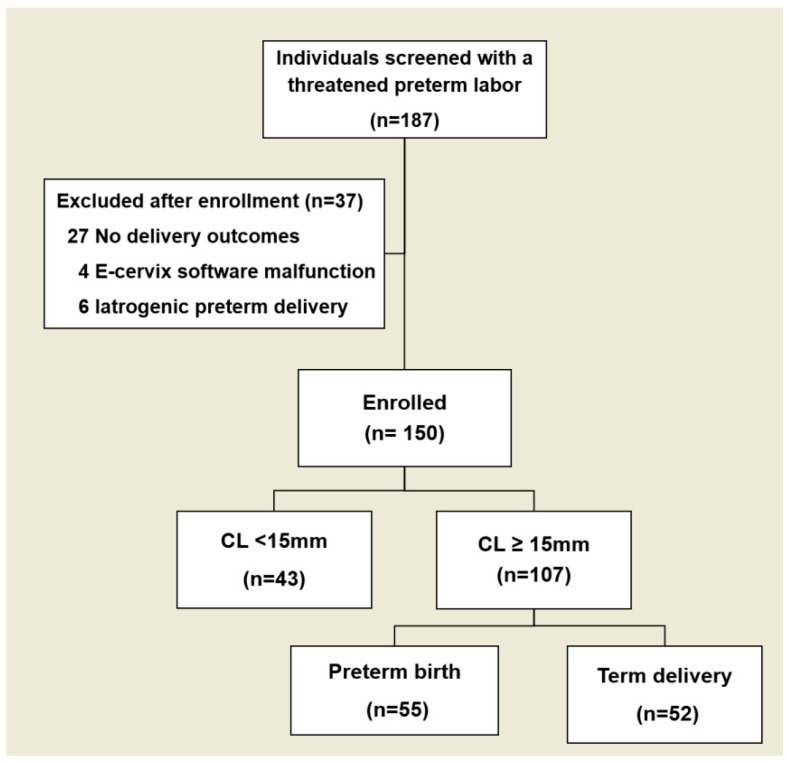
Flow diagram of enrollment and preterm birth outcome.

In a total of 150 women with threatened PTL, the time of examination averaged 28.96 ± 3.46 weeks of gestation. Of these, 84 (56%) had spontaneous preterm birth < 37 weeks, with 29 (19.3%) delivering before 34 weeks’ gestation. Upon enrollment, CL was significantly shorter in the preterm birth group compared to the term delivery group (*p* < 0.01), and funneling was more frequent in the preterm birth group (Table 1). E-Cervix parameters for each group are detailed in Table S1. We divided our original subjects into two groups: patients with CL < 15 mm and those with CL ≥ 15 mm. This division was intended to check if there are any differences in elastography parameters in relation to CL—whether the patient’s cervix is moderately short or extremely short as previously analyzed in patient with short CL [19]. In our study, there were no differences between patients with and without spontaneous preterm birth in terms of E-Cervix parameters, whereas CL itself demonstrated a significant difference in patients with CL < 15 mm (*n* = 43) (Table S2). In total of 107 women with threatened PTL and CL ≥ 15 mm were finally included in the analysis, following the exclusion of cases with extremely short CL (Figure 1).

Patients with spontaneous preterm birth had a significantly shorter CL (median, 21.9 [range (min–max), 15.0–41.8] vs. 27.5 [range, 15.0–45.0] mm; *p* < 0.01) than those with term delivery. Twin pregnancy was more frequent in the preterm birth group (32.7% vs. 11.5%, *p* = 0.01). All other characteristics were similar, including age, parity, history of prior preterm birth, use of tocolytic agents, and progesterone use (Table 2).

The E-Cervix parameters at the time of threatened PTL diagnosis for women with and without preterm birth are presented in Table 3. IOS, IOS/EOS ratio, and ECI in patients with spontaneous preterm birth were significantly higher than those in the term delivery group (*p* = 0.04, *p* = 0.02, and *p* = 0.04, respectively). Higher IOS and ECI mean that the cervical elasticity appeared softer and more heterogenous in women with threatened PTL and subsequent spontaneous preterm birth. Other comparisons, including EOS and HR, showed no significant differences between the two groups.

Multivariable logistic regression was performed only for parameters that were significantly associated with preterm delivery on univariate analysis. We also evaluated CL ≤ 25 mm as the current standard method (Table 4). A CL ≤ 25 m, a higher IOS/EOS ratio, and a higher ECI were significantly associated with an increased risk of preterm birth (adjusted odds ratio [aOR] 2.62; CI 1.17–5.89, aOR 10.46; CI 1.82–59.98, aOR 1.55; CI 1.01–2.37, respectively). Twin pregnancy and IOS were no significant association with an increased risk of preterm birth (*p* = 0.09, *p* = 0.18, respectively).

**Figure 2:**
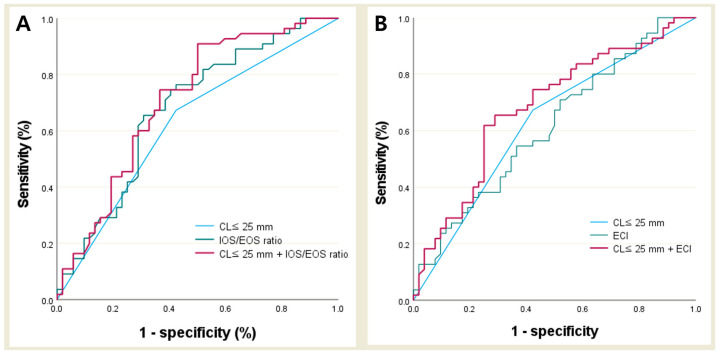
Receiver operating characteristic curve of predicting preterm delivery. (**A**): CL ≤ 25 mm, IOS/EOS ratio, and combination of CL ≤ 25 mm and IOS/EOS ratio. (**B**): CL ≤ 25 mm, ECI, and combination of CL ≤ 25 mm and ECI. CL; cervical length; IOS/EOS ratio, internal-to-external os stiffness ratio; ECI, elasticity contrast index.

To assess the predictive performance of E-Cervix parameters and CL ≤ 25 mm for spontaneous preterm birth, ROC analysis was performed in patients with threatened PTL and CL ≥ 15 mm. Upon ROC analysis, optimal cutoff values for predicting spontaneous preterm birth were determined based on the maximum Youden index, as detailed in Table 5. Among the evaluated parameters, the highest AUC was observed for the IOS/EOS ratio (AUC: 0.68, 95% CI 0.58–0.78, *p* < 0.01), followed by CL ≤ 25 mm (AUC: 0.63, 95% CI 0.52–0.73, *p* = 0.03), and ECI (AUC: 0.61, 95% CI 0.51–0.72, *p* = 0.04 yielded the highest Youden index (0.35), demonstrating a sensitivity of 65.5% and a specificity of 30.8%. Furthermore, the combination of IOS/EOS ratio and CL ≤ 25 mm exhibited superior predictive value for predicting spontaneous preterm birth compared to any of these parameters (CL ≤ 25 mm, IOS/EOS ratio or ECI) used alone (Figure 2).

## 4. Discussion

Women with threatened PTL are at an increased risk for preterm delivery. However, a significant clinical challenge persists due to the lack of specific diagnostic tools capable of reliably distinguishing between true preterm labor progressing to imminent delivery and threatened PTL that will not result in preterm delivery. Our findings indicate that E-Cervix parameters can be reliably measured and demonstrate significant predictive value for preterm birth in women with threatened PTL and a CL ≥ 15 mm at the time of diagnosis. Women who delivered before 37 weeks of gestation exhibited significantly softer internal part of the cervix, as indicated by higher IOS and IOS/EOS ratio, as well as greater heterogeneity of cervical elasticity (ECI). Specifically, we identified two E-Cervix parameters, the IOS/EOS ratio and ECI, that were significantly associated with preterm birth. Furthermore, the combination of CL ≤ 25 mm and IOS/EOS ratio exhibited superior predictive utility compared to either the IOS/EOS ratio alone or other individual parameters.

Threatened PTL is the most common reason for antepartum hospitalization in clinic fields, there have been many efforts to identify true PTL leading to preterm birth from false PTL. Waks et al. [25] reported that symptomatic sign with cervical changes is risk for preterm birth. Compared to symptoms alone or cervical findings such as shot cervix or dilated cervix alone, symptomatic with cervical finding group had higher preterm birth rate (11.2% vs. 49.0%), even than asymptomatic with cervical finding. Patients with a combination of symptoms and cervical findings threatening preterm labor. However, symptoms are too subjective and diverse to be used as objective predictive indicators. In addition, this report had limitation that previous preterm history and short cervix rate were higher in the symptomatic group for cervical findings; there is subject to potential selection bias, as 75% women diagnosed with a short cervix in the cervical finding group, while no women were diagnosed with a short cervix in the symptomatic group without cervical changes. Eroglu, D., et al. [25] reported that fetal fibronectin or phIGFBP-1 test in women with a short cervix could be used to predict spontaneous preterm delivery within 7 days and preterm delivery < 35 weeks. However, the value of these two tests lies mostly in their high negative predictive values (NPVs), while their PPVs are lower in women both with and without short cervixes, and they fail to accurately identify those who will ultimately deliver preterm regardless of cervical length. Fetal fibronectin is used for predicting preterm birth but varies according to gestational age at collection, population studied, prevalence of preterm birth, and single versus multiple screening [13]. The use of phIGFBP-1 may offer some advantages over fetal fibronectin, as it is not influenced by urine or seminal plasma contamination. Nonetheless, the presence of maternal blood can interfere with both tests, limiting their applicability in patients with active cervical bleeding. Lucovnik, M., et al. [15] reported that the EMG PV and PS peak frequency both identify true preterm labor more accurately with an ACU of 0.96. Despite this promising accuracy, EMG requires specialized equipment and lacks standardized protocols, leading to variability among studies. Moreover, its diagnostic reliability is further compromised by motion artifacts, fetal movements, and respiratory interference. Cervical elastography is a non-invasive, ultrasound-based method that offers several advantages over conventional techniques. Its compatibility with the transvaginal probe used for CL measurement facilitates seamless integration into clinical practice. This technique enables the continuous monitoring of cervical status through repeated measurements throughout pregnancy. Furthermore, cervical elastography can detect structural deformation prior to significant cervical shortening and provides objective, quantitative assessments of tissue stiffness. The inherent quantitative nature of its parameters contributes to enhanced reproducibility and reduced inter-observer variability, thereby establishing it as a more reliable diagnostic tool.

When examining prior research on the efficacy of cervical elastography in women with threatened PTL, Nazzaro et al. [23] reported distinct findings based on pregnancy type. In singleton pregnancies, they observed that a lower HR and significantly higher IOS and EOS were associated with preterm birth. In contrast, for twin pregnancies, Nazzaro et al. [21] found that women who delivered preterm exhibited only a significantly higher HR compared to those who did not deliver preterm.

In contrast to these findings, our study did not observe a significant difference in HR between the preterm birth and term delivery groups. This disparity may be attributed to differences in study populations and methodologies. Our study analyzed both singleton and twin pregnancies collectively and included only women with a CL ≥ 15 mm. On the other hand, Nazzaro’s studies included women with a CL of both above and below 25 mm, and reported that HR < 50% had diagnostic value. Specifically, the inclusion of patients with a very short cervix (less than 15 mm) in prior studies by Nazzaro et al. [21,23] potentially encompassed cases of incompetent internal os of cervix (IIOC), which might have influenced their observed HR values. Considering that a short cervix is a strong risk factor for preterm birth, and their finding of HR < 50% indicated relatively lower cervical stiffness compared to other cervical elastography studies, these differences in patient cohort composition could explain the discrepancy.

This study has several limitations. First, it had a relatively small sample size, underscoring the need for further research across diverse populations. Second, the absence of universally standardized elastography parameters and cutoff values remains a major challenge. To date, there is no consensus on optimal elastography parameters or specific cutoff values for reliably predicting preterm birth. This lack of standardization stems from variations in patient populations, methodologies, and the presence of differing cervical pathologies across studies, resulting in wide-ranging values and limiting the comparability of findings. Additionally, the predictive value of cervical elastography may vary by ethnicity and in women with underlying medical conditions, highlighting the necessity for more inclusive and representative studies.

Despite these limitations, our study offers several notable strengths. It was prospectively designed and conducted as a multicenter study, including both singleton and twin pregnancies, thereby enhancing its clinical relevance across a broader obstetric population.

Importantly, we found that the IOS/EOS ratio was the most useful parameter for predicting preterm birth in women with threatened PTL and a CL ≥ 15 mm. PTL is typically accompanied by cervical changes, such as funneling, which is associated with alterations in the IOS. However, as the absolute value of IOS can vary significantly between individuals, the IOS/EOS ratio provides a more reliable indicator of relative change. A higher IOS/EOS ratio reflects a softer internal os compared to the EOS, a finding consistent with cervical remodeling seen in PTL. Although there was no statistically significant difference in the sonographic detection of cervical funneling at the time of threatened PTL diagnosis, a softer internal os may still confer a greater risk for cervical change. The IOS/EOS ratio, therefore, may serve as a more stable marker of early cervical remodeling than the absolute IOS value alone. Furthermore, our study exclusively included women with a CL ≥ 15 mm. In cases of a moderately or severely short cervix, it can be difficult to fully represent the entire cervix due to the narrow region of interest and challenges in distinguishing between reference and target tissues. By focusing on this specific population, we minimized confounding factors such as IIOC, active labor, and extremely short cervices, allowing for a more accurate assessment of cervical elastography as an independent predictor in threatened PTL.

## 5. Conclusions

In conclusion, our findings suggest that cervical elastography with E-Cervix™ system offers a valuable tool for identifying women at higher risk for preterm birth in the context of threatened PTL, especially when cervical length is not markedly shortened. Specifically, women with a higher IOS/EOS ratio are at an increased risk of preterm birth. Given that early diagnosis is crucial for the effective treatment and prevention of preterm birth in PTL, utilizing E-Cervix to identify these higher-risk women can empower clinicians to develop personalized strategies for preterm birth prevention. This approach could potentially lead to a reduction in preterm labor rates and/or allow for the more efficient management of hospital resources.

## Figures and Tables

**Table 1:** Demographic information, clinical course, and delivery outcomes in patients with threatened preterm labor by preterm birth outcome.

	Term Delivery (*n* = 66)	Preterm Birth (*n* = 84)	*p*-Value
Age (year)	33 (26–42)	34 (21–40)	0.64
nulliparity	44 (66.7)	50 (59.5)	0.40
Pre-pregnancy BMI	24.42 (18.31–31.30)	24.30 (17.62–35.71)	0.86
Smoking	0 (0)	1 (1.2)	1.00
Twin pregnancy	8 (12.1)	29 (34.5)	<0.01
Prior preterm birth	4 (6.1)	11 (13.1)	0.18
History of LEEP	0 (0)	5 (6.0)	0.07
DM	1 (1.5)	1 (1.2)	1.00
HTN	0 (0.0)	2 (2.4)	0.50
GDM	9 (15.0)	11 (13.3)	0.81
Preeclampsia	1 (1.7)	3 (3.6)	0.64
GA at exam (weeks)	29.0 ± 3.3	28.9 ± 3.6	0.86
CL at exam (mm)	25.4 (9.0–45.0)	18.7 (2.7–41.8)	<0.01
<15 mm	14 (21.2)	29 (34.5)	
15 mm–25 mm	22 (33.3)	37 (44.1)	
>25 mm	30 (45.5)	18 (21.4)	
Funneling	9 (13.6)	26 (31.0)	0.02
Cerclage after enrollment	0 (0)	2 (2.4)	0.50
Tocolytics after enrollment	58 (87.9)	78 (94.0)	0.25
Progesterone after enrollment	27 (40.9)	39 (47.0)	0.51
GA at delivery (days)	38.7 ± 1.0	33.8 ± 2.7	<0.01
Cesarean delivery	31 (47.0)	26 (31.3)	0.06

Data are presented as the median (range), mean ± SD or *n* (%); BMI, body mass index; LEEP, loop electrosurgical excision procedure; DM, diabetes mellitus; HTN, hypertension; GDM, gestational diabetes mellitus; GA, gestational age; CL, cervical length.

**Table 2:** Demographic information by preterm birth in patients with threatened preterm labor with cervical length ≥ 15 mm.

	Term Delivery (*n* = 52)	Preterm Birth (*n* = 55)	*p*-Value
Age (year)	32 (27–42)	34 (21–40)	0.46
Nulliparity	34 (65.4)	36 (65.5)	1.00
Pre-pregnancy BMI	24.35 (18.31–31.31)	24.03 (18.65–32.30)	0.82
Smoking	0 (0)	1 (1.8)	1.00
Twin pregnancy	6 (11.5)	18 (32.7)	0.01
Prior preterm birth	4 (7.7)	6 (10.9)	0.74
History of LEEP	0 (0)	3 (5.5)	0.24
DM	1 (1.9)	1 (1.8)	1.00
HTN	0 (0.0)	2 (3.6)	0.50
GA at exam (weeks)	28.6 ± 3.51	28.4 ± 3.81	0.95
CL at exam (mm)	27.5 (15.0–45.0)	21.9 (15.0–41.8)	<0.01
≤25 mm	22 (42.3)	37 (67.3)	
>25 mm.	30 (57.7)	18 (32.7)	
Funneling	4 (7.7)	10 (18.2)	0.15
Tocolytics after enrollment	46 (88.5)	50 (92.6)	0.52
Progesterone after enrollment	19 (36.5)	20 (37.0)	1.00
GA at delivery (days)	38.7 ± 1.05	33.9 ± 2.53	<0.01

Data are presented as the median (range), mean ± SD or *n* (%); BMI, body mass index; LEEP, loop electrosurgical excision procedure; DM, diabetes mellitus; HTN, hypertension; GDM, gestational diabetes mellitus; GA, gestational age; CL, cervical length.

**Table 3:** E-Cervix parameters for term delivery vs. spontaneous preterm birth in patients with threatened preterm labor with cervical length ≥ 15 mm.

Parameters	Term Delivery (*n* = 52)	Preterm Birth (*n* = 55)	*p*-Value
IOS	0.26 (0.17–0.45)	0.30 (0.18–0.50)	0.04
EOS	0.32 (0.19–0.56)	0.32 (0.12-0.51)	0.37
IOS/EOS ratio	0.84 (0.36–1.55)	0.99 (0.63–1.95)	0.02
ECI	3.33 (1.99–5.72)	3.62 (2.46–6.67)	0.04
HR	65.90 (27.78–77.42)	59.37 (24.90–87.65)	0.17

Data are presented as the median (range). IOS, internal os stiffness; EOS, external os stiffness; IOS/EOS ratio, internal-to external os stiffness ratio; ECI, elasticity contrast index; HR, hardness ratio.

**Table 4:** Multivariable analysis of variables, including cervical length and E-Cervix parameters, for prediction of preterm birth in patients with threatened preterm labor.

	*p*-Value	aOR	95% CI
CL ≤ 25 mm	0.02	2.62	1.17–5.89
Twin pregnancy	0.09	2.69	0.86–8.41
IOS	0.18	1.46	0.84–2.51
IOS/EOS ratio	0.01	10.45	1.82–59.98
ECI	0.04	1.55	1.01–2.37

aOR, adjusted odds ratio; CI, confidence interval; CL, cervical length; IOS, internal os stiffness; IOS/EOS ratio, internal-to external os stiffness ratio; ECI, elasticity contrast index, aOR: adjusted for cervical length.

**Table 5:** Diagnostic performance of cervical length and E-Cervix parameters for prediction of PTD in patients with threatened preterm labor with cervical length ≥ 15 mm.

	ROC/AUC	95% CI	*p*-Value	Cutoff	Sensitivity (%)	Specificity (%)	Youden Index
CL ≤ 25 mm	0.63	0.52–0.73	0.03		62.7	62.5	0.25
IOS/EOS ratio	0.68	0.58–0.78	<0.01	≥0.96	65.5	30.8	0.35
ECI	0.61	0.51–0.72	0.04	≥3.21	70.9	51.9	0.19
**Models for predicting preterm birth**							
CL ≤ 25 mm + IOS/EOS ratio	0.71	0.61–0.81	<0.01	≥0.87	73.0	54.5	0.41
CL ≤ 25 mm + ECI	0.68	0.58–0.78	<0.01	≥2.99	83.8	59.1	0.37

CL, cervical length; IOS, internal os stiffness; IOS/EOS ratio, internal-to external os stiffness ratio; ECI, elasticity contrast index.

## Data Availability

The data from this study will be made available by the corresponding authors upon request. Due to privacy and ethical restrictions, the data are not publicly accessible.

## Supplementary Materials

The following supporting information can be downloaded at: https://www.mdpi.com/article/10.3390/diagnostics15151934/s1, Table S1: E-cervix parameters for full-term births vs. spontaneous preterm birth. Table S2: E-Cervix parameters for term delivery vs. spontaneous preterm birth in patients with threatened preterm labor with cervical length < 15 mm.
