# Supplementary material for: Diagnostic Efficacy of Cervical Elastography in Predicting Spontaneous Preterm Birth in Pregnancies with Threatened Preterm Labor
Source: Diagnostics (Basel). 2025 Jul 31;15(15):1934. doi: 10.3390/diagnostics15151934 (PMC12346534; doi:10.3390/diagnostics15151934)
Supplement: Supplementary file 1 [file diagnostics-15-01934-s001.zip › diagnostics-3755525-supplementary.pdf]

Supplementary Materials

Table S1. E-cervix parameters for full-term births vs. spontaneous preterm birth.

**Parameters**	**Parameter Definition**	**No sPTD (n = 66)**	**sPTD (n = 84)**	***P*-value**
IOS strain	Average strain within the system-defined 1-cm radius semicircle at the internal os	0.28 [0.17–0.51]	0.30 [0.18–0.55]	0.20
EOS strain	Average strain within the system-defined 1-cm radius semicircle at the external os	0.32 [0.19–0.56]	0.31 [0.12, 0.52]	0.19
IOS/EOS ratio	Average strain at the internal os divided by the average strain at the external os	0.87 [0.36–1.55]	0.99 [0.63–1.95]	<0.01
ECI	Strain heterogeneity within the entire cervix	3.42 [1.98–5.72]	3.61 [1.84–7.00]	0.18
Hardness ratio	Percentage of total cervix area characterized as hard (strain value < 0.3)	62.4 [22.2–82.9]	60.9 [20.5–87.7]	0.57

Definitions modified from Patberg et al., 2021;the measures of tissue strain range between 0 and 1, with lower values indicating harder tissue; Data are presented as the median [range], *p* values less than 0.05 are shown in bold.

Table S2. E-Cervix parameters for term delivery vs. spontaneous preterm birth in patients with threatened preterm labor with cervical length <15 mm.

Parameters	Term Delivery (n = 14)	Preterm Birth (n = 29)	*p*-Value
IOS	0.34 (0.17–0.51)	0.28 (0.18–0.55)	0.28
EOS	0.33 (0.19–0.52)	0.30 (0.20- 0.52)	0.34
IOS/EOS ratio	0.99 (0.71–1.39)	0.99 (0.64–1.44)	0.42
ECI	3.99 (1.98–5.19)	3.38 (1.84–7.00)	0.80
HR	53.59 (22.22–82.92)	62.35 (20.49–80.22)	0.19

Data are presented as the median (range). IOS, internal os stiffness; EOS, external os stiffness; IOS/EOS ratio, internal-to external os stiffness ratio; ECI, elasticity contrast index; HR, hardness ratio.
